# Mind Maps: Processed as Intuitively as Thought? Investigating Late Elementary Students’ Eye-Tracked Visual Behavior Patterns In-Depth

**DOI:** 10.3389/fpsyg.2022.821768

**Published:** 2022-04-08

**Authors:** Emmelien Merchie, Sofie Heirweg, Hilde Van Keer

**Affiliations:** ^1^Department of Educational Studies, Ghent University, Ghent, Belgium; ^2^University of Applied Sciences, Bruges, Belgium

**Keywords:** eye tracking, visual behavior analysis, educational process mining, visual literacy, elementary education

## Abstract

In this study, 44 late elementary students’ visual behavior patterns when reading mind maps were investigated, more particularly, the intuitive processing nature of their visual characteristics, reading sequence and presentation mode (i.e., mind map before or after text). Eye-tracked data were investigated by means of static early attention and dynamic educational process mining (EPM) analysis and combined with learning performance and retrospective interview data. All students seem to struggle with the map’s radial structure during initial reading. Also, the picture’s position in the map diverts students from consecutively reading interconnected branches. EPM analyses revealed different reading patterns in proceeding reading behavior. Students receiving a text first, seem to grasp the radial structure slightly more and show higher information integration attempts. They also attained higher free recall and coherence scores. The study concludes with instructional design principles for urgently needed explicit visual literacy instruction in elementary grades.

## Introduction

**FIGURE 1:**
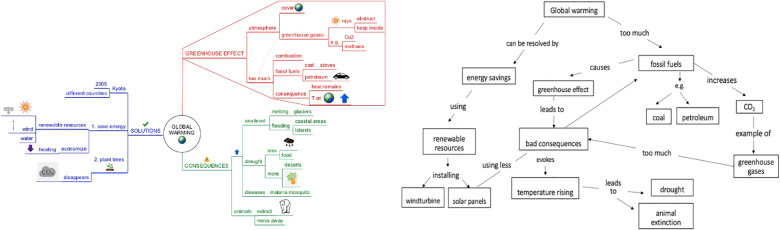
Difference between a mind map **(Left)** and a concept map (**Right**).

Understanding how visuals such as graphic organizers (GO) are processed and understood by learners is an important premise to effectively include them into study materials and classroom instruction (Schnotz et al., 2014). GO can be described as ‘spatial arrangements of words or word groups intended to represent the conceptual organization of text’ (Stull and Mayer, 2007; p. 810). The current study focusses on how the particular GO ‘mind maps’ is processed in late elementary education. Mind maps are colorful visuals including key words, images and symbols (Buzan, 2005). They align with the general definition of Stull and Mayer (2007) but differ greatly in their design and content arrangement from other GO, such as for example concept maps (Novak, 2002) (see for example Figure 1 illustrating the difference). In general, the use of GO to foster reading and learning is widely encouraged and scientifically underpinned by theoretical and empirical research studies (for an overview see Vekiri, 2002; Nesbit and Adesope, 2006). Briefly outlined, these theories refer to their advantages in decreasing students’ cognitive load (i.e., Cognitive Load Theory; Sweller and Chandler, 1994) and facilitating memory storage by the creation of a verbal and visual memory trace (i.e., Dual Coding Theory; Paivio and Clark, 1991). Also, recent empirical work keeps corroborating their effectiveness in reading and learning (Ponce and Mayer, 2014; Fiorella and Mayer, 2017; Ponce et al., 2019). However, an important premise to benefit from GO is being sufficiently competent to use them (Norman, 2012; Schnotz et al., 2014). For instance, for the proper comprehension visuals, a learner needs to establish links between the represented concepts on the different levels in the map, and their spatial representation (Körner, 2004). However, prior research (e.g., Körner, 2004; Herrlinger et al., 2017; Guo et al., 2018) indicates that various aspects can play a role in establishing proper links between concepts in a map. This study specifically focusses on (a) visual characteristics, (b) reading behavior and (c) the presentation mode (i.e., how map and text are combined) possibly playing a role in mind map processing.

First, as to their visual characteristics, mind maps (MM) in particular are being advocated as being very intuitive to use due to their visual characteristics (Buzan, 2005). Their radial structure would align with the brain’s neural network and its way of working. As shown in Figure 1 (left side), key words are placed on lines - called branches – leading out to associated super- and subordinate topics on subbranches. In this way, a mind map consists of different ‘levels’ radially spreading out (e.g., level 1 = greenhouse effect, level 2 = atmosphere, level 3 = earth cover, and so on). Furthermore, mind maps are visually enriched with pictures and colors, clustering information (Buzan, 2005). Indeed, various mind map characteristics are based upon brain and educational research confirming the positive learning effects of using association (Haber, 1970; Mento et al., 1999; Budd, 2004), mental imagery, colors (Anderson and Hidde, 1971), and gestalt principles such as the visual nearness of information (Wallace et al., 1998; O’Donnell et al., 2002). However, the effectiveness of these characteristics is underpinned by rather isolated studies, investigating not completely similar visuals, or only focusing on one particular design element (e.g., color). Conversely, MM can also be regarded as quit complex graphic organizers with dense information and a high intricacy. Because of this, researchers warn that young learners might experience cognitive overload (McTigue and Flowers, 2011; Guo et al., 2018).

Second, also a learners’ reading behavior can influence how links between concepts in a map are established. Commonly, it is advocated to initiate reading the upper-right page area of the mind map, an proceed in a clockwise way. For instance, in Figure 1, you start with the red branch reading ‘greenhouse effect’ and its associated branches reading from left-to-right and proceed in a similar manner for ‘consequences.’ However, to read the blue branch ‘solutions,’ (European) learners must change their conventional reading behavior, reading from right-to-left (e.g., from ‘solutions’ to ‘Kyoto,’ ‘save energy,’ etc.). The mind map structure, however, also allows other reading possibilities: you can screen the mind map vertically (e.g., reading ‘atmosphere,’ ‘too much,’ ‘sea level,’ ‘drought,’ ‘diseases,’ etc.), combine vertical with horizontal reading or first focus on the pictures (e.g., the polar bear). In contrast with concept maps, no arrowheads are provided in the map to guide readers visual reading behavior. In this way, a diversity of reading behaviors is possible. To date and to our knowledge, no research addresses how mind map are read and processed by learners and whether this influences their performance.

Third, research also points at the importance of considering the presentation mode of visuals, that is how text and maps are best introduced or sequenced to learners (e.g., Salmerón et al., 2009; Eitel and Scheiter, 2014; Herrlinger et al., 2017). On the one hand, it is advocated to present maps before the text because then they can serve as an organizing structure to assimilate text ideas (Mayer, 1979; Salmerón et al., 2009). However, this can also cause a ‘map shock’ whereby the specific organization and structure of the map evokes confusion (Goodnough and Woods, 2002). In this case, presenting the map after the text would be more beneficial (Herrlinger et al., 2017). Thus, research has been inconclusive when it comes to deciding how to best present (mind) maps to learners. Especially, when it comes to mind maps, no evidence-based guidelines are available concerning their presentation mode.

The above makes it questionable whether mind maps are indeed that intuitive to use. Their visual characteristics might possibly overload learners and they can be read in a variety of ways. Furthermore, also the presentation mode (i.e., presenting MM before or after the text) can influence students’ performance. Although mind maps have already been found to play a promising role in learning (Merchie and Van Keer, 2016a), the current knowledge base on students’ visual behavior patterns when studying mind maps is extremely limited. In this respect, research calls for identifying strategies when learner process specific graphic formats (Schnotz et al., 2014) and clarify the relationship with performance (van Amelsvoort et al., 2013). The aim of the present study is precisely to tackle this issue.

### Eye-Tracking in Graphic Organizers Research

The visual behavior pattern when reading mind maps has to our knowledge not yet been investigated. However, other researchers did already focus on how students’ study illustrated text (Jian, 2016; Mason et al., 2016; Jian and Ko, 2017), or spatial text representations (Nesbit et al., 2007; van Amelsvoort et al., 2013; Liu, 2014) by means of eye tracking. Eye-tracking is an online method, capturing an individual’s eye position and movements while viewing a certain stimulus (e.g., a text, picture, or GO) (Luo et al., 2014; Jian, 2016). This methodology is grounded in the eye-mind hypothesis (Just and Carpenter, 1980), stating that a student’s eye reveals not only where a student is looking at but also the viewer’s cognitive processing. Using eye tracking has the advantage of not interfering the learning process and the ability to collect several processing measures simultaneously (e.g., what students looked at, how long, sequences) with high temporal and spatial resolution (Hyönä and Lorch, 2004; Jian, 2016). With specific equipment, learner’s saccades (i.e., brief and rapid eye movements) and fixations (i.e., eyes resting on a part of the text) are tracked (Rayner, 1998; Penttinen et al., 2012).

**FIGURE 2:**
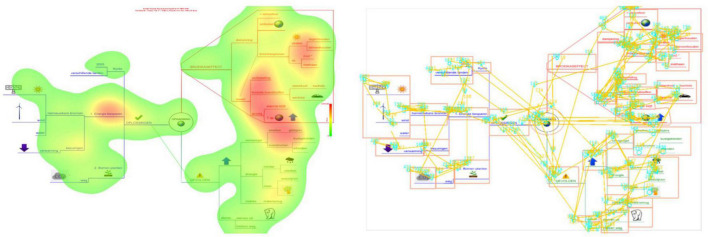
Example of a heat map **(Left)** and fixation and saccade map **(Right)**. The darker the area in the heat map **(Left)**, the more intensively students looked at that particular region in the mind map. Fixations in the saccade map **(Right)** are represented by circles, saccades by lines. Interest areas (IA) are represented by squares.

Afterwards, it is possible to analyze eye movement data and generate either static (i.e., heat, fixation and saccade maps, see Figure 2) or dynamic visualizations (i.e., gaze behavior videos or scan paths). For example, eye movement data analyzed in prior research were fixation duration (i.e., how long a particular area is visited) and run count (i.e., is how often a region is visited) (Dogusoy-Taylan and Cagiltay, 2014; Luo et al., 2014). As to the visualizations, heat maps visualize the regions students most intensively looked at, whereas fixations and saccade maps visualize where students focused on in the map (fixations) and which transitions (saccades) were made (see Figure 2). For instance, Liu (2014) inspected in saccade maps the direction of students’ eye-movement paths when receiving a concept map or not when reading text. More particularly, it was inspected whether paths were either more horizontally or vertically (top-down/bottom-up) to study students’ integration of concept map and text information. They found that participants receiving a concept map tended to skip over words in unimportant areas and using the concept map was effective in assisting students’ text-comprehension abilities (Liu, 2014).

Within the scope of this study, especially studying students’ dynamic visualizations is promising. Gaze behavior videos show how students’ eyes move from one spot to another when reading a mind map. This gives an indication of the nature and efficiency of processing (Jacob and Karn, 2003). However, analyzing dynamic visualizations is challenging and complex. Commonly, the visual is divided by the researcher into different Interest Areas (IA) (for an example, see the boxes in the right side in Figure 2). In this study, the duration, and the order in which these IA are visited are under investigation. Unfortunately, no-ready made method is available to analyze reading sequences in visuals (van Amelsvoort et al., 2013); van Amelsvoort et al. (2013) constructed eye movement sequence models (EMS-models) based on the occurrence of predefined sequences in table-like knowledge maps. However, due to the highly diverse reading possibilities of mind maps (see above), defining predefined sequences is very hard, making this data analyses technique less appropriate. Prior research has also used other data analysis techniques to investigate visual behavior patterns. For instance, to provide insights into the transitions of fixations some researchers focused on calculating transfer probabilities (Jian, 2016; Jian and Ko, 2017). This technique was applied to investigate how 10-year old students and adults read illustrated science text (Jian, 2016; Jian and Ko, 2017). Their results showed that it is possible to discern different reading behavior patterns between groups (i.e., young and adult learners, Jian, 2016; low and high ability groups; Jian and Ko, 2017). In this respect, the study of Jian (2016) illustrated that in general fourth-grade students’ visual literacy is limited. However, high ability groups did regress slightly more to previous paragraphs and transferred fixations back and forth between text and illustrations (Jian and Ko, 2017). Another approach to investigate students’ visual behavior patterns is to focus on students’ early attention. For instance, Nesbit et al. (2007) studied undergraduate students’ fixations in the few seconds a map was presented. They found that students first processed the maps by attending the nodes in the center, upper-center and upper-left regions. They discerned two processing approaches: a text-transfer model that adapts the highly practiced order for reading text. Here, students followed a left-to-right and top-to-bottom order. Other students followed a hub-first model, a strategy that gives early attention to highly connected, superordinate information, calling ‘hubs’ in their research (i.e., nodes with a relatively large number of links to other nodes). In a hub-first model, learner’ earliest fixations are in the central region of the concept map, and then processing order is biased toward nodes with a greater number of links. Their analyses were based on the median number of fixations before the first arrival at each node.

**FIGURE 3:**
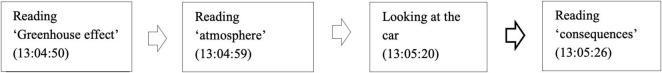
Example of a reading fixation pattern within a mind map.

The above-mentioned data analysis techniques applied in the research of Nesbit et al. (2007) and Jian (2016) are promising. However, most conducted research on this topic so far focused on the effect of drawings or text illustrations with a very limited number of Interest Areas (IA) under study (Jian, 2016; Jian and Ko, 2017). Studies undertaking the complex endeavor to study students’ visual behavior pattern in more complex GO, such as mind maps, are very scarce. An additional disadvantage of these techniques is that they rely on rather static information (i.e., fixation data such as run count) and thus apply mainly a variable-oriented approach. They do not provide us with detailed insights into the complex dynamics, or the exact order of proceeding executed activities (i.e., process-oriented approach). An innovative and promising technique in this respect is *educational process mining* (EPM), which can provide us with this kind of information (Bannert et al., 2014; Heirweg et al., 2020; Rogiers et al., 2020). Educational process mining permits to visualize complex processes on the base of moments in time where the activities have been executed (van der Aalst, 2011). This innovative data analysis technique was already successfully applied in prior research to study the cyclical nature of self-regulated learning processes (e.g., Bannert et al., 2014; Heirweg et al., 2020; Rogiers et al., 2020). Based on more than 100 think aloud protocols, Heirweg et al. (2020) visualized the process of text-learning and identified different patterns among high, middle, and low-achievers. Also, Rogiers et al. (2020) applied this technique to study how students with different learner profiles undertook a learning task. This methodology is also applicable on eye-tracking data, by extracting the start and end times of fixations in particular Interest Areas (see Figure 3 for an example). By means of EPM software, student data can be aggregated to generate the most frequently visited Interest Areas and paths between them. In the method section, this is explained in detail. Applying this innovative technique on eye tracking data could provide valuable additional insights into student’s dynamic mind map processing behavior. In this way, this research can be regarded as a pioneer in the application of EPM analysis on eye tracking data.

However, as with any methodology, some caveats are also related to the use of eye tracking and several researchers point at the added value of combining these metrics with think aloud or retrospective interviews to gain more insight into the reasons for specific processing behavior (Penttinen et al., 2012; Trevors et al., 2016; Catrysse et al., 2017). Although thinking aloud has been shown to be a successful methodology when investigating text learning, thinking aloud during working with mind maps appeared to be very difficult in late elementary education (Merchie and Van Keer, 2014). Therefore, the methodology of retrospective interviews was preferred in this research (van Amelsvoort et al., 2013) to complement the research findings.

### Aim of the Study

The general aim of this study is to map late elementary students’ visual behavior patterns when reading mind maps in light of text learning, and thus, to explore their intuitive processing nature. More particularly, it is investigated how learners process mind maps in terms of (a) their visual characteristics (e.g., attention drawn to pictures), (b) reading sequence (e.g., how are learners generally reading the mind map), and (c) presentation mode (i.e., does students’ visual behavior differ according to the mind map presentation, before or after a text). Furthermore, also the relationship with learning outcomes is explored. As research on complex visuals such as mind maps is scarce, it is difficult to put forward clear hypotheses underpinned by similar prior work. However, based on the theoretical framework, it could be assumed that (a) more attention is paid to mind map areas with pictures or higher information intricacy (Wallace et al., 1998; O’Donnell et al., 2002), (b) students generally will read the mind map in a clockwise way as advocated in practice, and (c) students receiving the mind map could possibly experience a ‘map shock’ by its specific organization and complex structure (Goodnough and Woods, 2002), which might in turn result in a more disorientated reading pattern when compared to students receiving a text first. However, based on memory presentation theories (e.g., Schooler, 2002) presenting a mind map before the text in turn might promote higher learning performance. It would allow students to make easier connections between the map, stored in memory as a holistic unit (Miller, 1956) and the text.

With this focus, this study addresses several limitations in previous research. First, the knowledge based on how elementary students process more complex visuals such as mind maps is extremely limited. Therefore, the present study targets late elementary grades, since their struggle with text reading and learning, making in-depth insights into this topic at this age pivotal (e.g., Merchie et al., 2014; Tielemans et al., 2016). It furthermore focusses on mind maps, an understudied visual in empirical research, although already frequently used in educational practice. Second, data analysis of dynamic visualizations such as gaze behavior videos is complex and challenging. Therefore, this study aims to combine different data analysis techniques (i.e., static and dynamic analysis on eye-tracked behavior, combined with performance and retrospective interview data) to answer the study’s research aim and provide rich valuable methodological and didactical insights for future research.

## Materials and Methods

### Participants and Design

A total of 44 elementary students aged between 11 and 12 years participated. 59,1% boys and 40,9% girls, 54,5% fifth graders and 45,5% sixth graders participated. For each student, the AVI-level^1^ was obtained from their teachers. Chi-square analyses indicated no significant differences between the conditions with regard to students reading fluency level (*X^2^* = 0.430, *df* = 8, *p* = 0.159). Furthermore, no significant differences were found between the conditions regarding students’ gender (*X^2^* = 0.802, *df* = 1, *p* = 0.52) and home language (*X^2^* = 0.366, *df* = 2, *p* = 0.366). Informed consent was obtained and none of the students were withdrawn by their parents from participation in the study. Students were instructed to study a mind map about global warming (see Section “Materials and Measures”). In the MMT- and TMM-condition students studied a 3-paragraph informative text of the mind map either before (MMT-condition, *n* = 23) or after text learning (TMM-condition, *n* = 21). In all conditions, the learning task was followed by a multilayered posttest and a retrospective interview (see Section “Materials and Measures”).

### Materials and Measures

#### Mind Map

**FIGURE 4:**
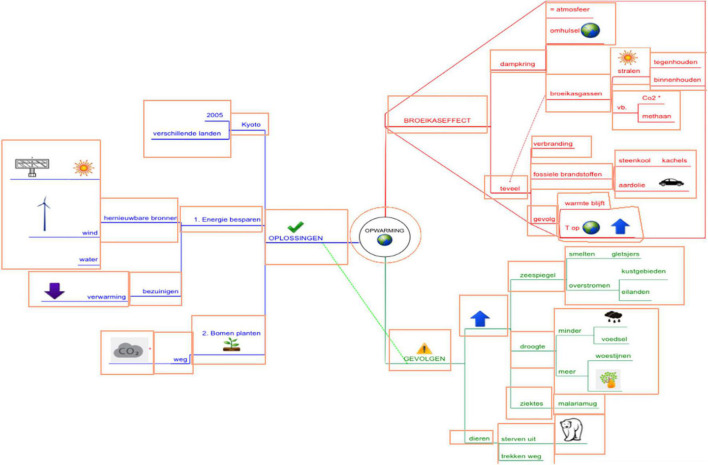
Original mind map (in Dutch) on global warming. In the result section, the mind map is translated into English. The boxes represent the Interest Areas (IA) and were not visible for the participants (see further).

**FIGURE 5:**
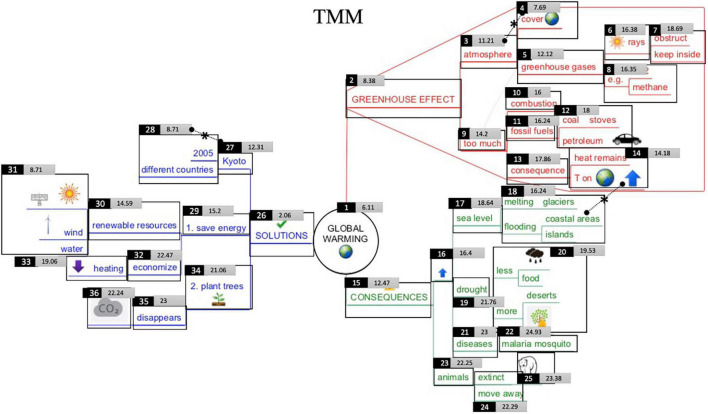
Visualization of students’ early attention in the Text-Mind Map condition (TMM). The smaller the number in the light gray rectangle, the earlier attention that particular Interest Area received in the first-pass reading process. Interest area number is located at the upper left of the IA. Level-1 branches = IA 2–15–26; level-2 branches = IA 3–9–16–23–27–29–34; level-3 branches = IA 4–5–10–11–13–17–19–21–24–28–30–32–35; level-4 branches IA 6–7–8–12–14–18–20–22–25– 31–33–36. Dotted lines with asterisks indicate significant differences in early attention between IA.

A computer-drawn expert mind map (Figure 4), constructed by a trained researcher was included in the experiment, consisting out of the central topic, 3 main branches (level 1-branches, e.g., ‘greenhouse effect,’ IA 2–15–26 in Figure 5), and related level-2 (e.g., ‘atmosphere,’ IA 3–9–16–23–27–29–34 in Figure 5), level-3 (e.g., ‘earth cover,’ IA 4–5–10–11–13–17–19–21–24–28–30–32–35 in Figure 5), level-4 subbranches (e.g., ‘sun rays’ IA 6–7–8–12–14–18–20–22–25–31–33–36 in Figure 5) (see Figure 5). The mind map was designed conform all specified effective characteristics in literature (e.g., color and radial structure; Buzan, 2005). Furthermore, the readability and data quality of the eye tracked behavior on the mind map was extensively pilot tested in advance. As auto-segmentation of Interest Areas (IA) was not possible for the mind map (e.g., due to integration as an image into the Experiment Builder Software), hand-drawn IA were assigned. It was verified that minimal IA were used that follow the perimeter of the object (Orquin and Holmqvist, 2019). In total 36 IA were defined.

#### Informative Text

**FIGURE 6:**
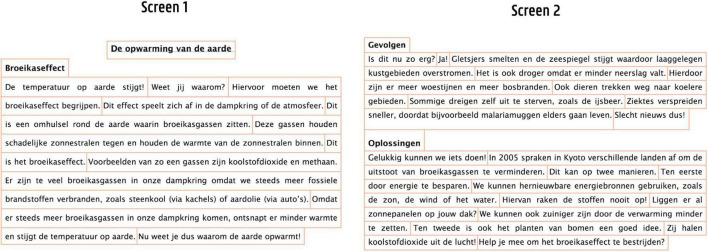
Informative text on global warming presented on two subsequent screens. The boxes represent the Interest Areas (AoI) and were not visible for the participants (see further). Since the focus of this study lies on the visual behavior pattern in the mind map, detailed analysis on the informative texts are not under investigation in this study.

The informative text consisted of a 316-word, 35-sentences informative text on global warming. The text contained three paragraphs that were presented on two subsequent screens (see Figure 6): the phenomenon of global warming (screen 1), the consequences and its solutions (screen 2). The text had a title (“global warming”) and each text paragraph was preceded by a subtitle (i.e., “greenhouse effect,” “consequences,” and “solutions,” respectively). The text was derived from a fifth-grade textbook and was already pilot-tested in prior intervention research. In addition, the overall text and the separate text paragraphs were analyzed by the Dutch Institute for Test Development (CITO) taking into account the lexical and sentence complexity. The overall text was found to be appropriate for this age group. Since the focus of this study lies on the visual behavior pattern in the mind map, detailed analysis on the informative texts are not under investigation in this study.

#### Prior Knowledge Measure

To assure that there was no large variation in students’ prior knowledge that might influence reading and learning (Dochy et al., 1999; Armand, 2001), students were asked to mention everything they already knew about global warming at a prior knowledge test. Students’ prior knowledge was rated per paragraph (i.e., greenhouse effect, consequences, and solutions) on a 4-point scale (0 = no prior knowledge, 1 = incorrect prior knowledge, 2 = partially correct prior knowledge and 4 = correct prior knowledge). Descriptive analyses indicate that students had little to no prior knowledge concerning the greenhouse effect (4,7% students had correct prior knowledge) and its solutions (3,1% students had correct prior knowledge). Slightly more students (23,4%) had correct prior knowledge concerning the consequences. Chi-square analyses indicated no significant differences between the conditions with regard to students’ prior knowledge (greenhouse effect: *X^2^* = 2.771, *df* = 6, *p* = 0.837; consequences: *X^2^* = 8.981, *df* = 6, *p* = 0.175; solutions: *X^2^* = 2.964, *df* = 4, *p* = 0.564).

#### Multilayered Posttest Measure

To promote reading-for-learning, a multilayered posttest measure was administered (Kim et al., 2018), consisting of three sub-tests which were answered verbally. First, students’ free text recall was measured by asking them what they remembered from the text. This test was administered orally, and students’ answers were recorded and transcribed afterwards. In line with other research (McNamara and Kintsch, 1996; Merchie and Van Keer, 2016a), students’ free recall was compared with the text content and a free recall score was calculated, representing the percentage of correctly recalled text information. In addition, also ‘free recall coherence’ was scored on a 4-point scale, indicating how coherently the recalled information was retold, ranging from ‘only words and short key sentences are mentioned without making inferences’ (e.g., ‘Animals extinct. We can plant trees’) to ‘key sentences or ideas are mentioned by making correct inferences’ (e.g., ‘A good idea is to plant trees because they filter carbon dioxide from the air’). Second, students completed seven multiple-choice questions each assigned 1 point when answered correctly. The generated questions assessed text learning at three levels of increasing difficulty (Korinth and Fiebach, 2018), reflecting knowledge that is either explicitly stated in the text (3 questions, e.g., ‘What is a different word for atmosphere?’), understanding associations between text elements (2 questions, e.g., ‘What is a bad example of a renewable energy source?’) and transfer questions (2 questions; e.g., ‘Does the greenhouse effect also take place in Belgium?’). Third, 5 open questions were administered. To answer these questions, students were required to make explicit inferences (scored in total on 13 points, e.g., ‘Why is it a shame that so many forests disappear because of the fires?’). Inter-rater reliability was high for the measures included in the multilayered posttest (κ_freerecall_ = 0.92, κ_coherence_ = 0.95, κ_multiplechoice_ = 1,κ_openquestions_ = 0.97).

#### Retrospective Interview

Students were shown a video of their gaze behavior produced by Data Viewer. The video shows a moving dot representing fixations points in the same speed as students processed the text (Catrysse et al., 2017). During the video replay, students were questioned about (a) how they dealt with the learning task (i.e., studying the text and/or mind map), (b) to describe and explain their reading behavior as reflected in their eye movements, thoughts, and beliefs that might have affected their learning, and (c) what they were thinking at that time. Leading questions were for example: How did you approach reading the mind map? Did you encounter difficulties? I see you paused here, is that correct? Can you explain why you are rereading this part of the mind map? Can you rephrase this branch in your own words? (Trevors et al., 2016). The retrospective interview took about 20 min.

### Eye Tracking

#### Eye Tracking Apparatus

The SR Eyelink Portable duo was used to record eye tracking data. This system includes a Host laptop and a Portable Duo USB camera, with a sampling rate up to 1,000 Hz binocular recording and accuracy typical between 0.25 and 0.5° in the head free-to-move mode. However, after pilot-testing the experiment (see further), it was decided to use a chin and forehead rest during the experiment. Resolution is <0.01° RMS error, perfect for microsaccade and pupil size measurement. The experiment was constructed and displayed using Experiment Builder software (SR Research, Canada) on a single laptop 15-inch screen, with a resolution of 2,280 pixels × 1,800 pixels.

#### Eye Tracking Procedure

Participants were individually seated in front of the display laptop in a dark and quiet room in their elementary school. All curtains were kept closed to avoid (changes in) natural sunlight and ensure data quality. Participants completed the prior knowledge measures and were provided with clear instructions, to get acquainted with the setting (Jian, 2016) and since task demands can influence their reading patterns (Kaakinen and Hyona, 2010; Ponce and Mayer, 2014). When students rephrased the assignment correctly, the student took place on an adjusted and comfortable chair, placed their head in the chin and forehead rest, which supported and stabilized students’ heads. This assured a constant distance of 60 cm between the screen and the students’ eye (cf., Catrysse et al., 2017; Korinth and Fiebach, 2018). The researcher initiated the formal experiment using the software Experiment Builder (SR Research, Canada) starting with a 9-point calibration and validation of the eye movements. After successful calibration, additional drift checks were performed to ensure identical start positions for the eyes during the onset of each task on the screen (i.e., before reading the instructions, before studying the text and before studying the mind map) (Kim et al., 2018). Text and/or mind map representation was manually initiated by the experimenter. The researcher and/or student could proceed to the next screen with a keypress. The abovementioned materials, measures and procedure was extensively pilot-tested with one fifth-grade boy and one sixth-grade girl. The main changes made after these pilot-tests included shortening the text length, simplifying and enlarging the mind map, finetuning instructions concerning calibration and using a chin and forehead rest. The experimental session including the learning task and multilayed posttest lasted on average 50 min per participant.

#### Eye-Tracking Data Selection

Data were processed and analyzed with the Eyelink Data Viewer 3.1.246 Software, SPSS and the Educational Process Mining software DISCO (see further). Prior to data export, data quality was verified (Orquin and Holmqvist, 2019). In total, data from eight participants was removed after visually inspecting scan paths indicating that fixations were not recorded reliably and accurately (e.g., due to sudden head movements after calibration). Afterwards and as advised for reading research, fixation cleaning was used, by means of a refined fixation cleaning algorithm (Data Viewer, 2018, p. 47; Orquin and Holmqvist, 2019). In particular, short nearby fixations were merged, fixations falling out of the interest areas were removed. Raw data were transformed into csv-files using DataViewer. These files were read into Excel for further analyses in SPSS and DISCO.

### Data Analysis

In order to conduct both static (Nesbit et al., 2007) and dynamic (EPM, Bannert et al., 2014; Heirweg et al., 2020; Rogiers et al., 2020) analysis, the eye tracking variables presented in Table 1 were extracted from Data Viewer in an Interest Area report. In line with Nesbit et al. (2007), we focused in the static attention analysis on the (order of the) fixations in the first few seconds after the map was presented (i.e., start and end time of the first run of fixations). For the static early attention analysis, the number of different Interest Areas visited so far before the first fixation was made into the current interest area was calculated across participants. The smaller the number, the earlier attention was paid to that particular interest area (Nesbit et al., 2007). Independent sample *t*-tests and one way analysis of variance were conducted to verify significant differences between early attention to different mind map regions (i.e., upper or lower half of the map), levels (see mind map description in ‘Materials and Measures’ Section), and between conditions. For the educational process mining (EPM) analysis, Event Sequence Data reports for all participants was extracted from Data Viewer. In this report, an overview is provided per participants of start and end times of the different visited Interest Areas during the complete reading process of the map (EyeLink Data Viewer 3.2.1., 2018).

**TABLE 1:** Extracted eye tracking variables in Data Viewer.

General information	Report and measure	Description
	Interest Area report: Trial_dwell_time	Duration of the trial (i.e., summation of all fixation durations)
	IA_run_count	Number of times the Interest Area was entered and left (runs)
Data analysis technique		
Static early attention analysis (e.g., Nesbit et al., 2007)	Interest Area report IA_First_fixation_visited_IA_count	The number of different interest areas visited so far before the first fixation is made into the current interest area. The smaller the number, the earlier attention was paid to that particular IA.
Dynamic educational process mining analysis (e.g., Bannert et al., 2014; Rogiers et al., 2020)	Event Sequence Data report: start time and end time of all visited IA	Overview per participant of the start and end times of all runs of fixations in the current interest area

#### Educational Process Mining

As already mentioned (see Section “Introduction”), EPM permits to create aggregated process maps visualizing students’ overall reading behavior. We were able to determine when, for how long and how many times Interest Areas were visited during the complete reading process by extracting the start and end times of all runs in the Event Sequence Data report (see Table 1). In line with prior research (Bannert et al., 2014; Heirweg et al., 2020; Rogiers et al., 2020) the fuzzy miner algorithm was used in EPM-analyses. In this algorithm, two metrics – ‘significance’ and ‘correlation’ – are used to calculate which activities (here: visited Interest Areas) and paths between them are included in the process models (Günther and Van Der Aalst, 2007). ‘Significance’ refers to the relative importance of visited IA and paths. The most frequent visited Interest Areas are retained in the model. ‘Correlation’ is used for selecting the paths. In this way, the software program DISCO automatically includes visited Interest Areas and paths between them that are often executed by a large group of students. Less frequent visited IA and paths, or paths that have been seldom conducted by only few students, are omitted (Günther and Van Der Aalst, 2007). The researcher can explore 1–100% of the visited Interest Areas and paths, ranging from displaying only the most frequent visited IA up to all visited IA, and from only the most domain paths to very seldomly occurring paths. By selecting a percentage of visited IA and paths (e.g., 50%), DISCO automatically calculates the activities and paths to be include in the map based on the algorithm mentioned above (van der Aalst, 2011).

To date, however, no specific standards are available on the amount of visited IA and paths to be included in the process models, and especially not when it comes to analyzing complex eye tracking data. This strongly depends on the type of research data and questions involved (Fluxicon, 2019). In general, the inclusion of as many activities (here: visited IA) and paths as possible while simultaneously avoiding too complex process models is recommended (Fluxicon, 2019). In the current study, the percentages of included visited IA and paths in the process models were carefully deliberated among 2 experts on self-regulated and visual literacy learning. First, all visited IA and paths were displayed, which resulted in extremely complex, uninterpretable maps. Second, gradually, the percentage of displayed visited IA and paths were decreased. The procedure was stopped when it resulted in the disappearance of all visited IA within a particular branch (e.g., all IA from the red, green or blue branch disappeared) and when important frequently occurring paths between IA for at least 50% of the participants became apparent. In this respect, 50% of the most frequent visited Interest Areas and 15% of the most frequently made paths between these IA were included in the analysis.

#### Multilayered Posttest

One-way analysis of variance was performed to examine differences in learning performance for the three different sub-tests of the multilayed posttest.

#### Retrospective Interviews

Retrospective interviews were transcribed verbatim and parsed into idea units. The units were thematically analyzed by means of the qualitative software package Nvivo 8 in two steps (Tabasso and van den Broek, 1985; Creswell, 2008; Mccrudden and Kendeou, 2014). First, all units were deductively analyzed by means of a coding scheme. This coding scheme is based on previously developed and theoretically underpinned assessment instruments for text-based learning (i.e., a think-aloud protocol coding scheme; Merchie and Van Keer, 2014; a task-specific self-report inventory; Merchie et al., 2014). The scheme reflects various cognitive (e.g., paraphrasing), metacognitive (e.g., scanning before reading and self-evaluation), motivational (e.g., revealing interest), text-learning strategies (e.g., asking self-made questions) students can apply during mind map and text learning. Second, codes were added deductively (e.g., adjusting reading pace). In total, 31% of the interviews, evenly distributed over the conditions, were double coded. The overall inter-rater reliability was measured using Miles and Huberman, 1994 formula (reliability = number of agreements/total number of agreements + disagreements). The interrater-reliability for the various coding categories was moderate to high, ranging between 77 and 100%. Due to the word limit and scope of this study, retrospective interview data was used here to complement our findings. Also, for this reason, only information on the main categories ‘reading sequence’ (i.e., receiving the mind map or text first), ‘planful approach’ (i.e., thoughtlessly, strategically, or driven by prior knowledge or curiosity) and ‘MM characteristics’ (i.e., perceiving the map as visual unit, pictures, colors, key words, and arrows) will be included. However, upon request, more detailed information on the retrospective interview data can be provided.

## Results

In total, independent sample *t*-test analyses indicate that students from the TMM condition (*M* = 121629,667 ms, *SD* = 51814,19381 ms) and the MMT condition (*M* = 156703,1667 ms, *SD* = 134128,488359 ms) spent roughly the same time [*t*(21.963) = 1.035 *p* = 0.312] on studying the mind map. On average, both conditions spent about 2 min reading-for-learning the mind map. It furthermore must be noticed that the total runt count (i.e., number of times an Interest Area was visited) is higher for the IA containing pictures [(*t* = 17,979) = −2,358, *p* = 0,030] (i.e., run count of IA without pictures: *M* = 3,26, *SD* = 1,03; and IA with pictures: *M* = 4,55, *SD* = 0,50.

### Static Early Attention Analysis

**FIGURE 7:**
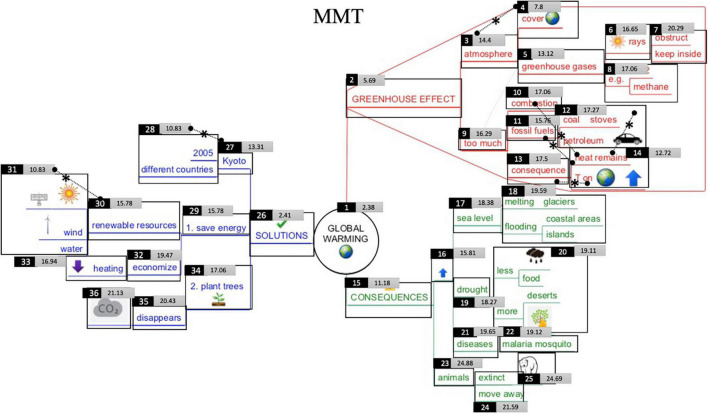
Visualization of students’ early attention in the Mind Map-Text condition (MMT). The smaller the number in the light gray rectangle, the earlier attention that particular Interest Area received in the first-pass reading process. Interest Area number is located at the upper left of the IA. Level-1 branches = IA 2–15–26; level-2 branches = IA 3–9–16–23–27–29–34; level-3 branches = IA 4–5–10–11–13–17–19–21–24–28–30–32–35; level-4 branches IA 6–7–8–12–14–18–20–22–25– 31–33–36. Dotted lines with asterisks indicate significant differences in early attention between IA.

To gain more insight into where students’ initial attention is drawn to when confronted with a mind map (e.g., visual characteristics such as pictures of areas with high information intricacy), Figure 5 (TMM-condition) and Figure 7 (MMT-condition) were created. The value in the center of each rectangle corresponds to the underlying Interest Area (IA) and represents the median fixations before first arrival at that particular Interest Area. The smaller the number, the earlier attention that particular IA received. Concerning the reading behavior, and bearing in mind the recommended ‘clockwise’-reading order for mind maps, it could be assumed that the IA in the red branch ‘greenhouse effect’ would receive earliest attention, following by the IA in the green branch ‘consequences’ and blue branch ‘solutions.’ Furthermore, it could also be assumed that, when following the prescribed left-to-right reading order within branches, students would give earlier attention to the IA more closely related to the main topic (i.e., level 1 and 2 branches) and then proceed to IA in the outer regions (i.e., level 3 and 4 branches) (e.g., for instance first reading ‘fossil fuels’ on level 3 and then ‘coal’ or ‘petroleum’ on level 4).

#### Across Conditions

When comparing the early attention given to the upper (IA 1 to 14 and 26 to 31) versus lower half of the map (IA 15–25 and 34–33), independent *t*-test analyses, indicate a significant difference in early attention to the information in the upper half of the map (*M* = 12.99, *SD* = 4.74) as compared to information in the lower half (*M* = 19.87, *SD* = 3.06) [*t*(34) = −5.026, *p* < 0.000]. This is also illustrated in Figures 5, 7: the smallest numbers are located at the center and upper half part of the mind map. Also, IA receiving latest attention in the initial reading process are indeed located on the lower half of the page. This is especially true for branches in the outer regions (i.e., level-3 and -4 branches in the outer side of the branch ‘consequences’ and ‘solutions’). One-way analysis of variance indeed confirm that, despite condition, there is a significant difference in early attention given to the four levels in the mind map [*F*(3) = 5.623, *p* = 0.003]. More particularly, *post hoc*-Tukey-tests show significant differences between level 1- (*M* = 7.06, *SD* = 4.79) and level 2-branches (*M* = 16.43, *SD* = 3.75) (*p* = 0.015), between level 1- and level 3-branches (*M* = 16.98, *SD* = 4.62) (*p* = 0.005) and between level 1- and level-4 (*M* = 18.05, *SD* = 3.84) branches (*p* = 0.002). There is no difference across conditions between early attention given to IA with (*M* = 14.42, *SD* = 6.49) and without pictures (*M* = 17.08, *SD* = 4.25) [*t*(34) = 1.495, *p* = 0.144].

#### Between Conditions

Very similar results occur between the two conditions. As to the early attention given to the individual Interest Areas, no significant differences between condition occur, except for the IA ‘malaria mosquito’ (IA 22) in the green branch [*t*(29) = −2,272, *p* = 0.031], receiving earlier attention in the MMT condition. No significant differences in early attention occur between conditions for early attention to upper regions [*t*(34) = 0.907, *p* = 0.371] and lower regions of the map [*t*(33) = −1.329 *p* = 0.193]. No significant differences between condition occur in early attention given to level-1 [*t*(34) = −0.949, *p* = 0.349], level-2 [*t*(27,154) = 0.524, *p* = 0.604], level-3 [*t*(34) = −0.922, *p* = 0.363] and level-4 branches [*t*(33) = −1.676, *p* = 0.103]. Also, no significant differences between conditions occur in early attention given to IA with pictures [*t*(34) = 0.107, *p* = 0.915] or without pictures [*t*(34) = 0.436, *p* = 0.666].

When inspecting Figures 5, 7 in more detail, two interesting aspects should additionally be noticed. First, some subordinate branches (i.e., level-3 branches) received earlier attention than superordinate branches (i.e., level-2 branches), although when following the presumed reading order described above, one would assume the opposite. For instance, from Figures 5, 7 it can be inferred that students gave earlier attention to the subordinate level-3 branch ‘cover earth’ (IA 4) than the preceding superordinate level-2 branch ‘atmosphere’ (IA 3). The same accounts for other level-2 and level-3 branches (e.g., IA 13–14 and IA 30–31): each time the level-3 branches receive earlier attention and often it contains a picture.

When statistically verifying this by means of a paired-sample-*t*-test within each condition, the differences in early attention between level-2 and level-3 IA are significant in the MMT-condition for IA 3–4 [*t*(12) = 2.322, *p* = 0.039], IA 13–14 [*t*(12) = 2.353, *p* = 0.036], IA 27–28 [*t*(12) = 2.857, *p* = 0.014], and IA 30–31 [*t*(17) = 2.385, *p* = 0.029]. Differences are significant in the TMM-condition for IA 3–4 [*t*(13) = 2.197, *p* = 0.047] and IA 27–28 [*t*(12) = 2.665, *p* = 0.021]. In Figures 5, 7, these significant differences are indicated with a dotted line and asterisk.

Second, also some lower-lying branches receive earlier attention than upper-lying branches. Here, one would also assume the opposite when following the presumed reading order of a mind map. For instance, in Figures 5, 7, the red level-4 branch ‘heat remains’ with an earth globe and an arrow (IA 14) received earlier attention than upper-lying branches (IA 12). The IA 11–12–13–14 can also be considered as a mind map area with high information intricacy. When verifying significant differences in early attention given to upper- versus lower-lying branches in the MMT-condition, significant differences are found between IA 10–14 [*t*(14) = 2.272, *p* = 0.039], IA 11–14 [*t*(16) = 2.289, *p* = 0.036], and IA 12–14 [*t*(14) = 2.769, *p* = 0.013]. In the TMM-condition, significant differences are found between IA 12–14 [*t*(16) = 2.389, *p* = 0.030]. This is also indicated by a dotted line and asterisk in Figures 5, 7.

### Dynamic Analysis: Educational Process Mining

**FIGURE 8:**
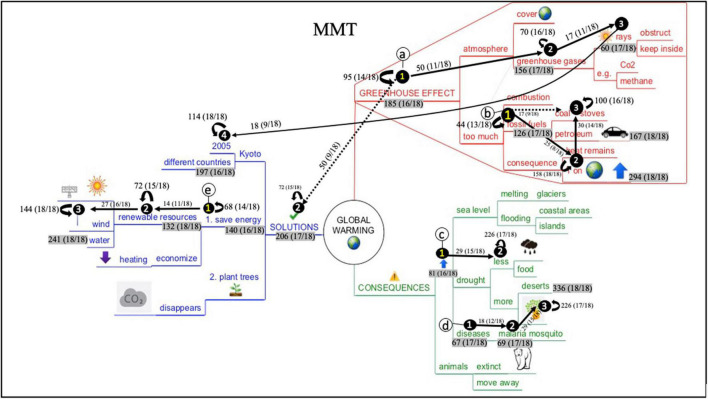
Aggregated process map of the Mind Map-Text (MMT) condition students.

**FIGURE 9:**
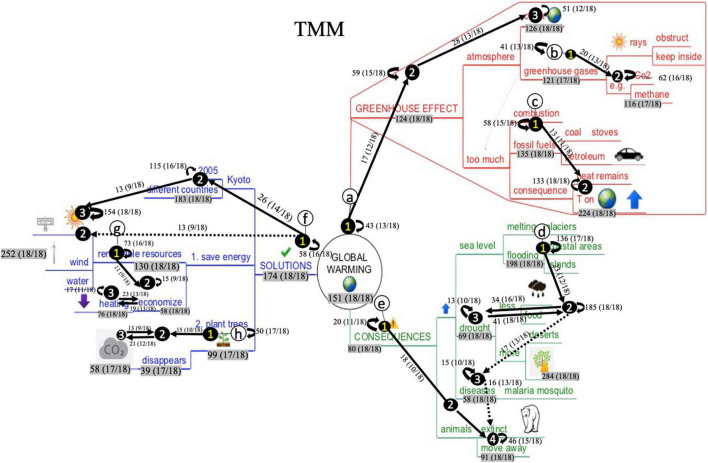
Aggregated process map of the Text-Mind Map (TMM) condition students.

Even though the static early attention analysis informs us on students’ initial visual behavior, no information is provided on particular reading sequences during the whole study process. To gain more insight into this matter, educational process mining analysis (EPM) were conducted. Therefore, aggregated process maps were generated - visualizing the most frequently visited Interest Areas and paths between them (see “Materials and Methods” section for a detailed description). Figures 8, 9 represent the aggregated process maps of the Text-Mind Map (TMM) and Mind Map-Text (MMT) condition students, respectively. In each of the maps, frequently occurring reading patterns are displayed (indicated with circled letters).

**FIGURE 10:**
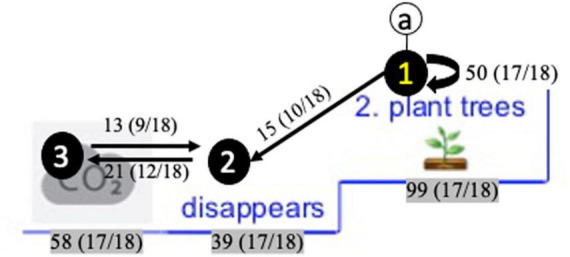
Snapshot of a reading pattern in the blue branch ‘solutions’ to illustrate its’ interpretation.

As interpreting these maps might seem complex at first sight, we first provide general information for correct map interpretation with a snapshot of one particular reading pattern in Figure 10. The reading pattern displays several elements: (1) circled alphabetic letters, (2) circled numbers on IA, (3) paths between the circled numbers, (4) loops (i.e., recurring arrows next to the IA), (5) numeral information accompanying IA, arrows, and loops. Figure 10 displays ‘reading pattern a.’ Circled numbers and paths in ‘reading pattern a’ display a frequently occurring reading sequence. For instance, in Figure 10, a right-to-left reading sequence was followed (i.e., from the branch ‘planting trees’ to ‘disappears’ and ‘CO_2_’). This means that – overall – students respected the prescribed reading order of this blue subbranches. Between the IA, either unidirectional or bidirectional arrows are displayed. This indicates whether the IA were read in consecution (unidirectional path) or in alternation (bidirectional path). In Figure 10, an alternation occurs between ‘disappears’ and ‘CO_2_,’ indicating that students frequently switched between these IA. Further, loops indicate that IA were visited several times in succession. In Figure 10, this is the case for the IA ‘planting trees,’ showing that this IA was successively reviewed in alternation by various students. The numeral information accompanying the elements display the frequencies of occurrence and – between brackets – the number of students that visited the IA at least once. For instance, during the whole reading process, the transition from ‘planting trees’ to ‘disappears’ was made 15 times by 55,5% of the students (10/18). The IA ‘planting trees’ was reviewed 50 times in alternation by 94,4% of the students (17/18), and the IA ‘CO_2_’ was visited 58 times in total 94,4% of the students (17/18).

As already mentioned, Figures 8, 9 contain different reading patterns. This means that, for both conditions, no single consecutive reading pattern could be identified by means of the EPM analyses. Consequently, students vary greatly in their mind map processing behavior. However, EPM analyses did reveal different reading sequences that were regularly executed by at least 50% of the participating students. These reading patterns and the differences between the two conditions are described below. A particular reading pattern stops at the moment that a large variety occurred in the way students proceeded their reading from that particular IA. Below, the reading patterns for both conditions are described.

In the aggregated process map of the TMM-condition students (Figure 9), eight frequently occurring reading patterns could be identified. Reading patterns are marked with the circled letters ‘a’ to ‘h’ (Figure 9). First, reading pattern ‘a,’ ‘e,’ and ‘f’ show similar characteristics: readers radiate out from the middle of the map, this is from the main branches to the superordinate and subordinate branches. However, it must be noticed that important ‘sublevels’ are skipped (e.g., students are skipping ‘atmosphere’ in the red branch, ‘animals’ in the green branch, and ‘Kyoto’ in the blue branch). Second, also reading pattern ‘b’ and ‘c’ show similarities. Both are top-down oriented, to a nearby diagonally underlying branch. Reading pattern ‘d’ in the green branch is remarkably initiated from the outer region, where students proceed in a top-down way. The largest part of the students (16/18) reconnects to the horizontally situated branch ‘drought,’ whereas 13 of the 18 students further proceed in a top-down way. Reading pattern ‘g’ and ‘h’ in the blue branch mainly follow the prescribed reading order, namely reading the branches subsequently from right-to-left. What furthermore must be noticed is the presence of three bidirectional pathways in this aggregated map (i.e., in reading pattern ‘d,’ ‘g,’ and ‘h’), indicating that students frequently switched between these IA. Also, the high number of loops for particular IA must be noticed, for instance for ‘heat remains, T on earth is rising’ (red branch), ‘melting glaciers, floods’ (green branch), ‘less food, more forest fires’ (green branch), and ‘2005 and different countries’ (blue branch) and all renewable recourses (blue branch).

In the aggregated process map of the MTT-condition students, 5 frequently occurring reading patterns could be identified. These are marked with the circled letters ‘a’ to ‘e’ in Figure 8. In ‘reading pattern a,’ students are reading horizontally, and bottom-up (to ‘sun rays’). Interestingly, half of the students proceeded consecutively to the upper section of the blue branch. Reading pattern ‘b’ and ‘d’ show similarities, in a way that students here seem to apply a slightly reverse clockwise approach in reading. It must be noticed that reading patterns ‘a,’ ‘b,’ and ‘d’ all show this bottom-up reading approach to a diagonally upper lying branch. In reading pattern ‘c,’ 15 of the 18 students skip an intermediate level (i.e., ‘drought’). Reading pattern ‘e’ is characterized with the prescribed right-to-left reading order. Interestingly here is that no bidirectional pathways occurred. However, also here, some frequently occurring loops were present, for ‘heat remains, T on earth is rising’ (red branch), ‘less food, more forest fires’ (green branch), ‘2005 and different countries’ (blue branch), and all renewable recourses (blue branch).

### Learning Performance

One-way analyses of variance were performed to analyze learning performance differences between the conditions. With regard to the free recall score, significant differences were found between the conditions [*F*(2,63) = 13.072, *p* < 0.001]. More particularly, *Post Hoc* pairwise tests with Bonferroni correction indicate that TMM-condition students attained significantly higher learning performance than the MMT-condition students (*p* = 0.003). This is also the case for the ‘free recall coherence’ score [(*F*(2,63) = 7.512, *p* = 0.001], were *Post Hoc* pairwise tests indicate that TMM-condition students attain significantly higher scores than the MMT-condition (*p* = 0.003) students. No significant differences were found between the conditions with regard to the multiple-choice test score [*F*(2,63) = 0.275, *p* = 0.76] and cued recall test score [*F*(2,63) = 1.192, *p* = 0.331].

### Retrospective Interview

Students were also retrospectively interviewed about the sequence in which they read the mind map and reasons behind their reading behavior. In total, 14 students experienced difficulties to articulate why they started reading in a particular region (*n*_TMM_ = 5, *n*_MMT_ = 9). Other students referred to several reasons for paying earlier attention to particular regions, for instance: interest (*n* = 4) (e.g., “I was interested in what happened with the sea level,” “I immediately wanted to know all the solutions”), information intricacy (*n* = 6) (e.g., “I started in the blue branch, because it seemed more simple,” or the other way around “I started in the red branch, because it seemed most difficult”), visual characteristics (*n* = 7) (e.g., “I started in the red branch, it was the biggest branch,” “I choose blue, my favorite color,’ “because I liked the polar bear”), or linking with prior knowledge (*n* = 4) (“I saw the different animals, I also have animals at home,” “I recognized the solar panels, we also have that on our roof,” “because I already knew that planting trees was a solution”). Some students also mentioned why their attention was drawn to a particular Interest Area. For instance, IA containing number (e.g., ‘2005,’ and ‘1. Save energy’) because “they looked important” of they assumed there was a particular reading order. Three students in the MMT-condition referred to negative aspects when being initially confronted with the mind map and ‘stress’, being overwhelmed with all the difficult words.

As to their subsequent reading behavior, students also revealed several reasons to skip or pay extra attention to particular information. In this respect, MMT-conditions students refer to the pictures’ attractiveness as a reason for paying less attention to the words. Some students in the TMM-condition (*n* = 2) also explicitly referred to the pictures as memory anchor. Students in both conditions also indicate that they skip information they already know. However, when the researcher inserted some comprehension checks, it became apparent that their assumed prior knowledge was not always correct. Further, both conditions also revealed experienced difficulties with particular elements in the map (*n*_MMT_ = 10, *n*_TMM_ = 9). Both conditions referred to the unclearness of particular images (such as the ‘T’ for temperature, the picture of the forest fire or the meaning of ‘CO_2_’ in the cloud). MMT-condition students also experienced more difficulties with particular terminology (e.g., greenhouse effect, Kyoto, methane). Both conditions were also asked for advice to optimize the mind map and mentioned providing more explanation on how to read the mind map, providing a legend with more information on the included difficult words, symbols, abbreviations and pictures, asking students for advise on to be included images, and adding more space between the branches.

## Discussion

### Discussion of the Results

This study investigated the so-called intuitive processing nature of mind maps. More particularly, it was investigated how learners process mind maps in terms of their visual characteristics (e.g., pictures and information intricacy), reading sequence (e.g., radial or clockwise reading order), and presentation mode (i.e., mind map presentation before or after a text). Furthermore, also the relationship with learning outcomes was explored. To this aim, students’ eye-tracked data was analyzed by means of a static early attention analyses (i.e., variable-oriented approach), dynamic educational process mining analyses (i.e., process-oriented approach) combined with learning performance and retrospective interview data.

First, static early attention analyses revealed that the mind map’s visual characteristics and radial structure are not that self-evident for students, since – regardless of the condition – students in their earliest attention seem process the map by focusing on the upper half-region. It thus seems that they initially apply a text-transfer approach as described by Nesbit et al. (2007), following the highly practiced order of text reading (i.e., starting upper-left and reading from left to right, top-to-bottom). Also, some significant differences were found concerning early attention given to level-2 and level-3 branches, and to upper-versus lower-lying branches in the map. As most of these Interest Areas integrate pictures, numbers or high information intricacy, the map’s visual characteristics seem to play a role in attracting students’ attention, though possibly not always in a constructive way. For instance, when students pay earlier attention to subordinate branches containing pictures (i.e., level-3 branches) they might neglect important information on the superordinate levels (i.e., level-2 branches). This was also mentioned in the retrospective interview data, wherein students mentioned several reasons for starting in a particular map region (e.g., interest, information intricacy, linking with prior knowledge, etc.). However, skipping important superordinate levels might have detrimental effects on students’ map comprehension. In this respect, Schnotz and Bannert (2003) already warned for the integration of pictures and their possible negative effects on the construction of a coherent mental text model. This study also indicates that the integration and especially the location of pictures within (mind) maps must be deliberately thought out (e.g., for instance, by integrating more pictures on superordinate levels in the map). Further, although linking with prior knowledge is considered to be an important reading and learning strategy (Weinstein and Jung, 2011), it also entails pitfalls. Students might rather quickly read over areas on which they believe to have already sufficient prior knowledge on, even though the opposite could be true. Future data collection on a larger sample and more in-depth retrospective interviews should shed more light on how precisely the integration of pictures leads students’ attention and how prior knowledge might affects readers’ eye gaze (Amadieu et al., 2009). Furthermore, also other aspects of mind maps such as higher information intricacy in certain map areas warrant further investigation.

Dynamic EPM analyses provided us with more detailed insights in learners’ proceeding reading behavior. A first aspect that became apparent was the very large variety in students’ visual behavior patterns. It was consequently impossible to identify one consecutive visual behavior pattern for all students. This lies in contrast with findings of prior EPM research where overall, one study pattern for (groups of) learners could be identified (Bannert et al., 2014; Heirweg et al., 2020; Rogiers et al., 2020). However, we did manage to discern frequently occurring reading patterns. First, more frequently occurring reading patterns could be identified for the TMM-condition, indicating slightly lower reading variability within this condition. This might be due to the preceding text, which might have served as a sort of internal organizing structure for students to assimilate text ideas (Mayer, 1979; Salmerón et al., 2009), or the ‘map shock’ students’ in the MMT-condition might have experienced (Goodnough and Woods, 2002) leading to more disoriented reading behavior. Second, and in contrast with the MMT-condition students, more reading patterns radiating out from the middle of the map were identified in the TMM-condition, indicating that – despite their initial struggle – they more seem to grasp the radial structure of the map in their subsequent reading behavior. Also, more bidirectional paths were included in the TMM-condition students’ process map. This might indicate that they have attempted to a higher degree to integrate information (Alemdag and Cagiltay, 2018). Dynamic EPM analyses also revealed that both conditions seem to focus intensively on Interest Areas containing pictures during the whole reading process. Although one could assume that students are still attracted to these pictures or might be using them as a memory anchor, retrospective interview data revealed students’ struggle with correctly comprehending them. Further, students in both conditions do not seem to struggle with changing their conventional reading behavior in the left part of the map (i.e., reading from right-to-left).

As to learning performance, it was expected that MMT-condition students would attain performance scores, given the easier to be made connections between the map, stored in memory as a holistic unit (Miller, 1956) and the text (e.g., Schooler, 2002). Against expectations, a learning effect considering recall performance was found in favor of the TMM-condition students, however, only for the free recall test and their recall coherence. Here, possibly, the mind map might have played a fundamental different role in knowledge acquisition in both conditions, consequently being subject to different kinds of processing (Schnotz et al., 2014). In the TMM-condition, the preceding text might have been used for coherence-oriented general processing, that is guiding the learner’s conceptual analysis resulting in a more coherent network and an initial mental model. Afterwards, a more task-driven selective processing might have been used (i.e., on demand, as easily accessible external representations for updating the mental model). However, future research should study more in-depth the mental model construction and the way a mind map played a fundamental role in it (Schnotz et al., 2014) and investigate the relationship between students’ graphical and reading comprehension (Mayer and Gallini, 1990; Hannus and Hyönä, 1999; Roberts and Brugar, 2014). Further, the lack of significant differences between conditions for the multiple-choice test and cued recall might be due to the experiment’s duration. In this respect, prior meta-analysis revealed that more effective learning outcomes were found the longer a learner utilized the map (e.g., Schnotz et al., 2014). In our study, students in both conditions only spend about 2 min on average studying the map.

In sum, the applied data analysis techniques point at both similarities and differences in students’ mind map processing approaches. As to the similarities, results indicated that all students – regardless of the condition - do seem to struggle with some essential mind map characteristics such as grasping the radial structure in initial reading, subsequently reading the branches from super- to more subordinate levels, and correctly interpreting integrated pictures. However, also differences were shown in favor of students receiving the text first, as discussed above. Still, overall results show students’ struggle with the so-called intuitive nature of mind maps and their rather visual illiterateness to correctly read and interpret information in the maps. This is a striking finding, since they are used to frequently in practice and confirms that indeed, visual literacy instruction only received only scant attention in practice so far (Guo et al., 2018).

### Limitations and Directions for Future Research

Also, some limitations and directions for future research should be addressed. First, the applied methodology overcomes difficulties with digital pens and the think aloud methodology, used in prior research (Merchie and Van Keer, 2014). Analyzing eye-tracked data uncovers important online processing behavior and provided us with insights into the obstruction students experience when being opposed to visuals such as mind maps. However, students were not able to interact with the study materials. In line with prior research (Jian, 2016), we wanted to avoid the cognitive coast of switching between two sources (text and mind map). However, investigating in-depth how learners integrate information when text and mind map are presented simultaneously should be a following step (Guo et al., 2018). Moreover, a large diversity of eye tracking metrics can be generated from eye-tracking experiments. In this respect, well-considered choices must be made by the researcher. Additional in-depth analyses on particular eye tracking metrics retrieved from a larger sample could shed further light on the topic under investigation (e.g., fixation duration, first- and second-pass reading time, number of fixations; Rayner, 2009; Catrysse et al., 2017; Jarodzka and Brand-Gruwel, 2017). Additionally, also caveats of the educational process mining (EPM) methodology have already been described in prior research (Bannert et al., 2014; Heirweg et al., 2020; Rogiers et al., 2020). A disadvantage of this technique is that it currently lacks the possibility to statistically test differences of process mining models between conditions or relate this to other outcome measures such as learning performance (Bannert et al., 2014; Heirweg et al., 2020). We would highly welcome future research to resolve this important issue. Further, as to the aggregated nature of the educational process mining analysis, it might be possible that important individual differences are omitted. Therefore, future research is encouraged to engage in in-depth qualitative analyses. For example, in the research of Luo et al. (2014) researchers classified complete replays of students studying a matrix into three categories: (1) topical (i.e., largely scanning from one idea to another within a topic), (2) categorical (largely scanning from one category to another), (3) random when no discernable pattern occurred. In the research of Merchie and Van Keer (2016b) four different mind map elaboration approaches were discerned (i.e., how students construct their mind map, that is either inconsiderately, associatively, structured, or categorically). It might be investigated if these approaches could also be transferred to processing approaches. Additionally, prior research also showed that students with higher reading ability allocate more attention to crucial concepts (Amadieu et al., 2009). It is therefore very worthwhile in future research to investigate more in-depth how particular student characteristics (e.g., prior knowledge, cognitive, reading or spatial abilities, having particular learning difficulties) might influence the successful study of spatial word arrangement such as (mind) maps. Also, these kinds of individual characteristics should be investigated in future research. Furthermore, an important difference with prior EPM research applies to the granularity of the displayed activities (here: visited Interest Areas) and paths. For the eye-tracking data in this study, the displayed percentage of paths had to be decreased to 15% to get interpretable results (in contrast with prior EPM-research displaying 33% of the paths; e.g., Rogiers et al., 2020). This once again illustrates that standards for EPM-analyses set in a particular study context cannot simply be transferred to other research, and the decision on the percentage of included activities and paths largely depends on the investigated process (Fluxicon, 2019). Future eye-tracking research applying the EPM analyses is therefore strongly advocated to replicate our study and broader our insights.

This study concludes by stressing its innovative nature and suggesting some didactical guidelines for practice. This study is highly relevant since insights into students’ initial competences to effectively use visuals such as mind maps in reading and learning is currently lacking. However, without well-honed visual literacy skills, students are unlikely to employ visuals to their fullest potential (Guo et al., 2018). In this respect, the current research is innovative in two ways. First, by its focus on the initial and developing visual literacy competences of late elementary students. More particularly the educational relevant competence of being able to effectively use visuals such as mind maps when reading-for-learning is investigated in young learners. Therefore, the topic of this study can be situated at the crossroad of research domains such as educational psychology, subject didactics, educational design, visual and disciplinary literacy and inform these disciplines, respectively. Also, authentic learner materials were used, in contrast with other research focusing on one graphic and one accompanying text block (Guo et al., 2018). Second, the methodologically applied perspective in this study is diverse and innovative. More particularly, static early attention analysis, dynamic educational process mining analyses on online eye-tracking data, offline performance tests and retrospective interviews were combined (i.e., variable- and process-oriented approach). Particularly, to our knowledge, EPM has never been applied on eye-tracking data, especially not studying such a large number of interest areas under investigation, compared to other research (e.g., Liu, 2014; Jian, 2016). In this respect, we want to inform the research community on the possibilities of this promising technique. However, applying this technique also entails several challenges, as described above. In this respect, to broaden our insights, we strongly encourage future research to further explore EPM on eye-tracked data, also in larger samples and explore the possibilities of including statistical testing between models.

To conclude, this research provides opportunities to develop more evidence-based didactical guidelines for visual literary instruction when working with visuals such as mind maps. In this respect, Renkl and Scheiter (2017) discern between material- and learner-oriented interventions. Material-oriented interventions are related to reducing the complexity of the visual. It this respect, it is very important to consider the visual’s complexity in advance, since researchers have shown that often, visuals are not rigorously selected or pilot tested, resulting in putting the interpretational burden on students (Roberts and Brugar, 2017). Attuned to our study results, it might be interesting in this respect to include particular perceptual cued in mind maps guiding students reading behavior to point to critical information for facilitating reading (e.g., including arrowheads or numbers) (van Amelsvoort et al., 2013). Second, also the location of the pictures in the map should be rethought, since they naturally draw student’s attention. Therefore, it is advocated to include more picture on superordinate levels. Learners in our study also advocate to include small legends underneath the map, clarifying briefly used abbreviations, pictures or difficult words. Third, it is also advised – when used in combination with a multi-paragraph informative text – to first present the text and the mind map afterwards. Potentially, the mind map could also be digitally presented in animated format, to reduce its’ visual complexity. However, Schroeder et al. (2018) advocate more research on investigating possible benefits of static versus animated maps.

Learner-oriented interventions are related to training of learning prerequisites, training or prompting, which is also essential since prior research revealed that teachers rarely teach how to interpret visuals (Coleman et al., 2011). In this respect, there is an urgent need for instructional practices that better scaffold students to develop and optimize their visual processing skills (Guo et al., 2018). In this respect, our research also shows that is important to build sufficient prior knowledge on mind map conventions (i.e., how to read the maps’ radial configuration, why particular elements are presented in the mind map). In this respect, for instance, van Amelsvoort et al. (2013) advise to introduce learners to follow different phases in the study of visuals, for instance (1) reading the headers, (2) reading the body by following perceptual cues or glancing over the diagram to get the initial cues, (3) reading the diagram based on the content, and (4) studying the diagram in other directions as well. Here lies a promising role for eye movement modeling examples (EMME) and online modeling explicit strategy instruction (Jarodzka et al., 2013; Mason et al., 2017). By means of eye-tracking, an expert learner could model how mind maps are best processed. The model can, for instance, focus on crucial elements identified in our study (e.g., radial structure, subsequently reading the main and subbranches etc.). In addition, and in line with other researchers (Metros, 2008; van Amelsvoort et al., 2013; Schnotz et al., 2014), we argue that visual literacy instruction should be part of teachers’ pre- and in-service professional development since learners are extensively trained to read text, but hardly in diagrams of visuals in schools.

## Data Availability Statement

The raw data supporting the conclusions of this article will be made available by the authors, without undue reservation, to any qualified researcher.

## Ethics Statement

Ethical review and approval was not required for the study on human participants in accordance with the local legislation and institutional requirements. This study conforms to the General Ethical Protocol for Scientific Research at the Faculty of Psychology and Educational Sciences of Ghent University (version 2017-May 2018). There was an active written informed consent from the participants’ school principal. Passive informed consent was obtained from the participants’ parents.

## Author Contributions

EM was in charge of setting up the experimental design, data collection, and writing the main part of the manuscript. SH provided in-depth assistance throughout the data analysis (process mining in particular). All authors contributed throughout the different writing stages of the manuscript.

## Conflict of Interest

The authors declare that the research was conducted in the absence of any commercial or financial relationships that could be construed as a potential conflict of interest.

## Publisher’s Note

All claims expressed in this article are solely those of the authors and do not necessarily represent those of their affiliated organizations, or those of the publisher, the editors and the reviewers. Any product that may be evaluated in this article, or claim that may be made by its manufacturer, is not guaranteed or endorsed by the publisher.

## Footnotes

1 In Flanders, the AVI-index (i.e., Analyse Van Individualiseringsvormen [*Analysis of Individualization Forms*]) is used to indicate students’ reading fluency level. The index contains twelve difficulty levels, starting with ‘AVI-start’ being the easiest to ‘AVI-PLUS’ being the most difficult.
